# *Emiliania huxleyi* endures N-limitation with an efficient metabolic budgeting and effective ATP synthesis

**DOI:** 10.1186/1471-2164-15-1051

**Published:** 2014-12-02

**Authors:** Sebastian D Rokitta, Peter Von Dassow, Björn Rost, Uwe John

**Affiliations:** Alfred Wegener Institute - Helmholtz-Centre for Polar- and Marine Research, Am Handelshafen 12, Bremerhaven, 27570 Germany; Facultad de Ciencias Biológicas, Pontificia Universidad Católica de Chile, Edificio Nuevo N° 210, Santiago, Chile; Instituto Milenio de Oceanografía, Concepción, Chile; CNRS, Sorbonne Universités UPMC Univ. Paris 06, Pontificia Universidad Católica de Chile, Universidad Austral de Chile, UMI 3614 Evolutionary Biology and Ecology of Algae, Santiago, Chile

**Keywords:** *Emiliania huxleyi*, Limitation, Malate:quinone-oxidoreductase, Metabolism, Microarray, Nitrogen, Ornithine-urea-cycle, Polyamines, Proline oxidation, Transcriptomics

## Abstract

**Background:**

Global change will affect patterns of nutrient upwelling in marine environments, potentially becoming even stricter regulators of phytoplankton primary productivity. To better understand phytoplankton nutrient utilization on the subcellular basis, we assessed the transcriptomic responses of the life-cycle stages of the biogeochemically important microalgae *Emiliania huxleyi* to nitrogen-limitation. Cells grown in batch cultures were harvested at ‘early’ and ‘full’ nitrogen-limitation and were compared with non-limited cells. We applied microarray-based transcriptome profilings, covering ~10.000 known *E. huxleyi* gene models, and screened for expression patterns that indicate the subcellular responses.

**Results:**

The diploid life-cycle stage scavenges nitrogen from external organic sources and -like diatoms- uses the ornithine-urea cycle to rapidly turn over cellular nitrogen. The haploid stage reacts similarly, although nitrogen scavenging is less pronounced and lipid oxidation is more prominent. Generally, polyamines and proline appear to constitute major organic pools that back up cellular nitrogen. Both stages induce a malate:quinone-oxidoreductase that efficiently feeds electrons into the respiratory chain and drives ATP generation with reduced respiratory carbon throughput.

**Conclusions:**

The use of the ornithine-urea cycle to budget the cellular nitrogen in situations of limitation resembles the responses observed earlier in diatoms. This suggests that underlying biochemical mechanisms are conserved among distant clades of marine phototrophic protists. The ornithine-urea cycle and proline oxidation appear to constitute a sensory-regulatory system that monitors and controls cellular nitrogen budgets under limitation. The similarity between the responses of the life-cycle stages, despite the usage of different genes, also indicates a strong functional consistency in the responses to nitrogen-limitation that appears to be owed to biochemical requirements. The malate:quinone-oxidoreductase is a genomic feature that appears to be absent from diatom genomes, and it is likely to strongly contribute to the uniquely high endurance of *E. huxleyi* under nutrient limitation.

**Electronic supplementary material:**

The online version of this article (doi:10.1186/1471-2164-15-1051) contains supplementary material, which is available to authorized users.

## Background

Phytoplankton is responsible for about 50% of the global primary production, driving the global carbon cycle and sustaining the oceans’ ecosystems [1]. The majority of the open-ocean areas are generally limited in the macronutrients N and P, and primary production is sporadically fuelled by eddy-mediated upwelling of remineralized nutrients [2]. Scenarios of global change predict a warming of the sea-surface and concomitantly a stronger stratification of water masses. Open-ocean upwelling events could thereby decrease in frequency and intensity, lowering nutrient supply to surface waters and ultimately productivity [3]. In coastal areas, in contrast, intensified eastern boundary current upwelling and anthropogenic eutrophication are predicted to increase nutrient levels, enhancing growth of microalgae [4, 5]. Thus, changing nutrient regimes may become even stronger regulators of primary productivity and potentially alter the ecological and biogeochemical functions of these marine habitats.

Coccolithophores belong to the phylum Haptophyta, evolutionarily deep-branching photosynthetic eukaryotes that arose from a secondary endosymbiosis and are distinct from the red/green and brown lineages with respect to their phylogeny, physiology and cell architecture [6, 7]. The numerically most abundant coccolithophore in the modern ocean, *Emiliania huxleyi*, is a common constituent of phytoplankton assemblages from tropical to sub-polar oceans and is able to form large monospecific blooms up to the polar fronts [8]. These calcifying phytoplankton not only fix carbon into biomass but also into calcite and therefore contribute significantly to the organic as well as the inorganic marine carbon pumps. *Emiliania* is thus an important regulator of the relative vertical fluxes, which influence the CO_2_ uptake capacity of the oceans [9]. The global existence of diverse strains or ‘ecotypes’ of this species has led to the recognition of a ‘species-complex’, i.e., a large group of organisms, which share common ‘core’ parts of their genomes but possess individual genetic material that reflects their ecological niche differentiation [7, 10].

The ability to tolerate enduring limitation in macronutrients N and P seems to enable certain members of this species-complex to thrive in oligotrophic environments [7, 11]. The responses of *E. huxleyi* especially to N-limitation have been subject to numerous studies, typically showing that under N-limitation, growth rate ceases and the calcite to organic carbon ratio increases [12], photosynthetic performance declines [11] and that photosynthate exudation can dominate over biomass production [13, 14]. However, underlying physiological mechanisms of phytoplankton responses to macronutrient limitation remain largely unknown.

*E. huxleyi* furthermore exhibits a haplo-diplontic life-cycle: the diploid, calcifying cells can undergo meiosis and generate haploid cells [15, 16]. These non-calcified, motile haplonts can disperse and propagate independently until eventually -it is assumed- two individuals undergo syngamy and create a new, diploid cell. Because only the diploid stage is susceptible to lytic viruses, this life-cycling is thought to function as an important ecological escape strategy [17]. Indeed, laboratory studies have suggested that the stages exhibit pronounced physiological and transcriptomic differences, assumed to reflect the natural history of this species with ecological niche differentiation also between life-cycle stages [16, 18, 19].

We hypothesized that the haploid and diploid phases would show marked differences in their ability to resist N-limitation, as suggested by their distinct morphology and gene usage. Also, we hypothesized that the genes most prominently up-regulated in response to N-limitation are shared between life phases, reflecting more general biochemical requirements under nutrient stress.

Here, we report the cellular responses to N-limitation on the basis of macroscopic phenomenology and physiology (growth, elemental quotas, photosynthetic viability) as well as the underlying molecular processes (gene expression) in diploid and haploid life-cycle stages of *E. huxleyi*. These results can enable us to generalize and parameterize cellular functioning of protists under contrasting environmental conditions, a prerequisite not only for unravelling the genomic repertoire of this organism but also for a successful integration of this key phytoplankton group in models of global carbon cycling [20, 21].

## Results and discussion

### Growth characteristics and fluorometry

**Figure 1:**
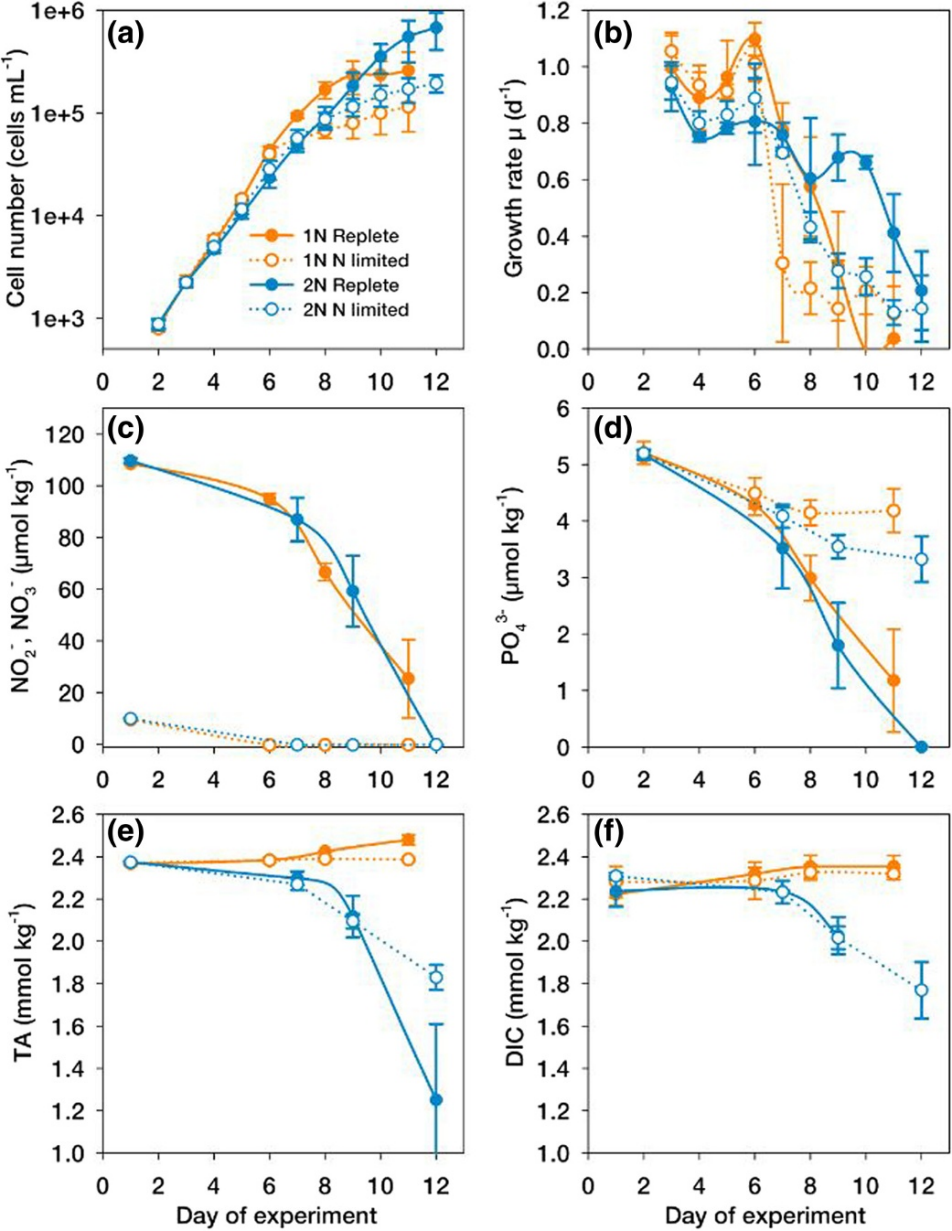
**Culture dynamics and seawater chemistry during the experiments with haploid (orange) and diploid (blue)** ***Emiliania huxleyi*** **grown with (N-replete, filled symbols) and without (N-limited, open symbols) NO**_**3**_^**−**^ **supplementation; (a) Cell concentrations; (b) Specific growth rates; (c) Concentrations of nitrite (NO**_**2**_^**−**^ **) and nitrate (NO**_**3**_^**−**^ **); (d) Concentrations of phosphate (PO**_**4**_^**3−**^ **); (e) Total Alkalinity (TA); (f) Dissolved inorganic carbon (DIC).** Error bars denote 1 SD (n = 3).

In the course of the experiment (Figure 1a), growth rates of diploid and haploid cells started in the range of 0.8 - 1.0 d^−1^ when nutrients were replete, but declined rapidly with the beginning of N-limitation at days 6 and 7 and approached zero around day 12 (Figure 1b). Nutrient analyses show that in the limited cultures, nitrate and nitrite (NO_3_^−^, NO_2_^−^) were fully depleted already at days 6 and 7 (Figure 1c), but phosphate (PO_4_^3−^) concentration was well above limiting levels (Figure 1d; [22]), proving that limitation in N, not P, caused the growth arrest.

To monitor carbon chemistry over the course of the experimental bloom, medium samples were analyzed for total alkalinity (TA) and dissolved inorganic carbon (DIC; Figure 1e,f). In the diploid *E. huxleyi* cultures, TA decreased over the course of the experiment due to calcification. The concomitant drop in DIC, despite re-equilibration with ambient air, is attributed to the decrease in TA because this parameter determines the capacity of DIC uptake [23]. In the non-calcifying haploid cultures, TA was slightly elevated due to the consumption of NO_3_^−^ [23]. Consequently, continuous aeration led to slight increases also in DIC towards the end of the experiment. TA and DIC were used to calculate [CO_2_] and pH (Additional file 1: Figure S1a,b), proving that over the course of the experiment, [CO_2_] was always >20 μmol kg^−1^, i.e., more than sufficiently high so that co-limitation of N and C can be ruled out. Also the calculated pH ranged within a band of 7.85 ± 0.15 over the whole experiment indicating that DIC was successfully replenished in the cultures selected for evaluating the effects of N-limitation. Hence, it can be stated that DIC and particularly CO_2,_ were not limiting over the course of the experiment and that the observed phenomena stem from N-limitation rather than from carbon limitation.

In consequence, the typical responses to N depletion [8] were seen from the cellular elemental quotas (Additional file 2: Figure S2a-e), i.e., an increase in particulate organic carbon (POC) content, decreases in cellular particular organic nitrogen (PON) content and drastically elevated POC:PON ratios when cells experienced N-limitation. Although the content in particulate inorganic carbon (PIC) was low due to the generally low pH [24], slight increases in cellular PIC content under N-limitation in the diploid stage were visible. This phenomenon is usually attributed to the reduction of biomass buildup due to limitation being stronger than the simultaneous reduction in PIC production [8].

**Figure 2:**
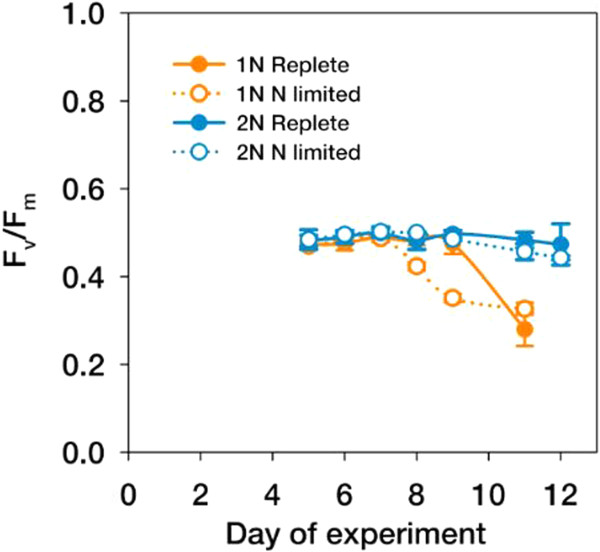
**Ratios of variable:maximum fluorescence (F**_**v**_ **/F**_**m**_ **) measured in haploid and diploid** ***Emiliania huxleyi*** **over the course of the experiment.** Color coding follows Figure 1. Error bars denote 1 SD (n = 3).

Fluorescence data showed that diploid *E. huxleyi* were viable in the exponential growth phase of the experiment (F_v_/F_m_ = ~0.5) and maintained photosystem functionality under N-limitation with only insignificant decreases in F_v_/F_m_ until the end of the experiment at day 12 (Figure 2). In the haploid life-cycle stage, in contrast, F_v_/F_m_ values dropped significantly after day 7, i.e., the onset of limitation, by up to 36% at day 11 (two-way ANOVA, followed by Holm-Sidak post-hoc test, *P* < 0.001; Figure 2). We caution that F_v_/F_m_ represents the maximal efficiency of PSII after dark adaptation. No statements can be derived about actual in-situ photosynthetic rates. Our data confirm the exceptional ability of diploid *E. huxleyi* to maintain functional photosynthetic electron transfer chains under N-limitation [11] and clearly demonstrate that the haplont lacks this capability.

### Transcriptomic response

**Figure 3:**
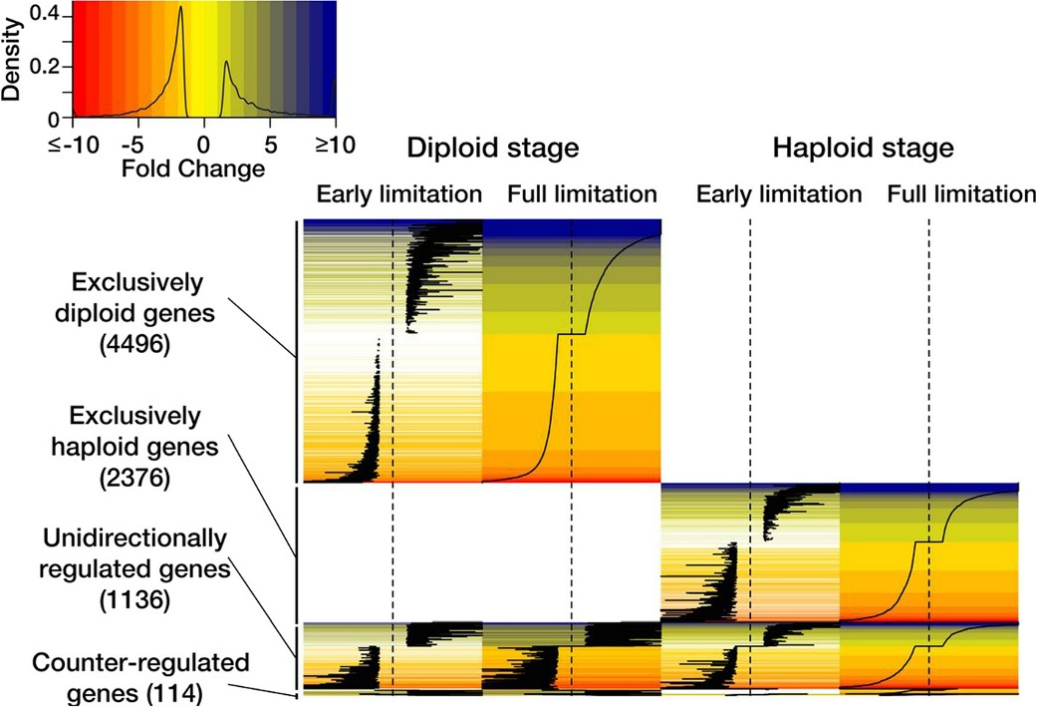
**Heat map representation of the observed transcriptomic responses to N-limitation in haploid and diploid** ***Emiliania huxleyi*** **.** The color-coding indicates ‘fold-change’ expression values between ≤ −10-fold (red) and ≥10 fold (blue). The density histogram reflects the data distribution, i.e. the frequency of observed expression ratios. In horizontal direction, the heat map depicts the time course of N-limitation in haploid and diploid individuals. In vertical direction, clusters of transcripts reflect the usage of genes, i.e. stage-specific, unidirectional, counter-regulated.

Mining the obtained microarray data revealed that distinct but functionally consistent sets of genes responded in haploid and diploid cells (Figure 3; Additional file 3: Figure S3; Additional file 4: Spreadsheet S4): Of all the ~28700 transcripts screened towards their reaction to N-limitation, 4496 genes (55% of all significantly regulated genes) changed expression only in the diploid stage. 2376 genes (29% of all significantly regulated genes) were differentially expressed only in the haploid stage. Only 1136 genes (14% of all significantly regulated genes) experienced unidirectional expression changes in both life cycle stages under full N-limitation. A residual of 114 genes (1% of the significantly regulated genes) showed a counter-regulation between stages. These results are consistent with a tripartite genome, where significant portions of genes show highly stage-specific expression patterns, as seen previously under replete growth conditions [16, 19].

The comparison of the two assessed time points ‘early limitation’ and ‘full limitation’ revealed that these two datasets are very coherent: ~60-70% of the genes of the ‘full’ response are already responding in the ‘early’ stage of limitation (Figure 3; Additional file 4: Spreadsheet S4). Those are indeed the most striking indicators for the cellular rearrangements reported in this study. This suggests that the cellular ‘first response’ to N-limitation is a rapid and comprehensive restructuring of the basic metabolism that occurs in an ‘all-or-nothing’ manner. At the later time point, we observed strong amplification of this ‘first response’ expression patterns, indicating that the triggered cellular program is gradually intensified with the time, or with the degree of starvation.

Under ‘full limitation’, we furthermore observed additional regulation of genetic machinery. It may be suspected that these genes are expressed due to the ongoing nutrient stress. However, the large number of ‘unknowns’ does not allow further statements on their specific function. Although we report the data for both time points in the supporting information spreadsheets we will, for simplicity, focus on the data of the ‘full limitation’ response as here the observed transcriptomic responses are more clearly depicted. To maintain scope and clarity, the transcriptomic responses to N-limitation of each stage are elaborated separately, beginning with the diploid calcifying stage. The exemplary transcript clusters that are explicitly mentioned in the text are collected in Additional file 5: Spreadsheet S5.

## Diploid stage

### Deactivation of protein biosynthesis

In the diploid stage, N-limitation caused significant up- and down-regulation of 2447 and 3307 transcript clusters. The dataset indicates a global decrease of cellular activity: Biosynthesis appeared de-activated at the levels of transcription and translation, as numerous features indicate down-regulation of RNA polymerases (RNA polymerase I subunits GJ20109, GJ07166, GJ20108; RNA polymerase II subunits GJ02734, GJ00400, GJ23980, GJ22750; RNA polymerase III subunit GJ01405) as well as ribosomes in the cytoplasm (40S ribosomal proteins GJ21301, GJ07497, GJ16295; 60S ribosomal proteins GJ03947, GJ23720, GJ08989) and the organelles (Mitochondrial/chloroplast ribosomal proteins GJ21760, GJ18064, GJ08340, GJ16340).

Likewise, most tRNA synthetases were concertedly down-regulated, indicating that cells decreased de-novo protein-synthesis. In line with this, we further observed strong down-regulation of machinery involved in the de-novo syntheses of amino acids (e.g., Asparagine synthase GJ02807, Aspartate kinase GJ09398), purines and pyrimidines (AICAR-transformylase/IMP cyclohydrolase GJ07918; Glycinamide ribonucleotide synthetase/Aminoimidazole ribonucleotide synthetase GJ13295; Adenylosuccinat-Lyase GJ10021, GJ05238; Dihydroorotase GJ11657, GJ20229; Glutamine phosphoribosyl-pyrophosphate amidotransferase GJ02409; Phosphoribosylformylglycinamidine synthase GJ05919, GJ00085). These expression patterns strongly suggest that the overall biosynthetic activity is to a considerable extent regulated at the transcript level and is strongly reduced in response to N-limitation.

### Deactivation of the light reactions

The dataset indicated a strong down-regulation of numerous critical components of the photosystems (e.g., PS I reaction center subunit IV GJ17955; PS II 12 kDa extrinsic protein GJ10857; PSII reaction center GJ09667; PS II oxygen-evolving enhancer protein GJ19198) and the light-harvesting antennae (e.g., fucoxanthin-chlorophyll a-c binding protein GJ02563: chloroplast light harvesting protein isoform 2 GJ23738) as was previously observed under nutrient limitation [25]. Also, elements of the xanthophyll cycle (zeaxanthin epoxidase GJ10268, GJ00453, GJ22205; violaxanthin deepoxidase GJ02541, GJ07458), and plastidic ATP synthesis (GJ15617, GJ19940) were prominently down-regulated, indicating that the photosynthetic light reactions were strongly decreased. Cells down-regulated the expression of crucial enzymes of chlorophyll synthesis e.g., glutamate-semialdehyde aminomutase (GJ01010), and Mg-chelatase (GJ00007, GJ01253), and thus use ancient regulatory switches that are well conserved even in land plants [26]. Despite this overall down-regulation of light-harvesting capacity, cells prominently induced fucoxanthin-chlorophyll a-c binding proteins of the LI818-group (GJ26033, GJ04295, GJ16863) under full N-limitation. These proteins have been shown to be expressed under high light conditions and are believed to function in the mitigation of photo-oxidative stress [27]. Their prominent expression supports the notion that cells make attempts to reduce overall light harvesting and reductant input under N-limitation. Apparently this strategy is successful, as the residual photosystems showed no signs of extensive light stress or compromised electron flow, as F_v_/F_m_ values remained high (Figure 2).

The down-regulation of several plastidic triose and hexose translocators (GJ03350, GJ07072, GJ01768) indicated decreased transport processes between the plastid and cytosol. Apparently, this large-scale plastid-inactivation not only minimizes carbon and reductant input into the cell, but also requires the reallocation of plastid-based biochemical tasks, like N-assimilation, to the cytosol, leading to an overall metabolic isolation of the chloroplast. In this metabolic low-throughput mode, however, cellular N must be efficiently recycled intracellularly.

### Reconstellation of nitrogen metabolism

**Figure 4:**
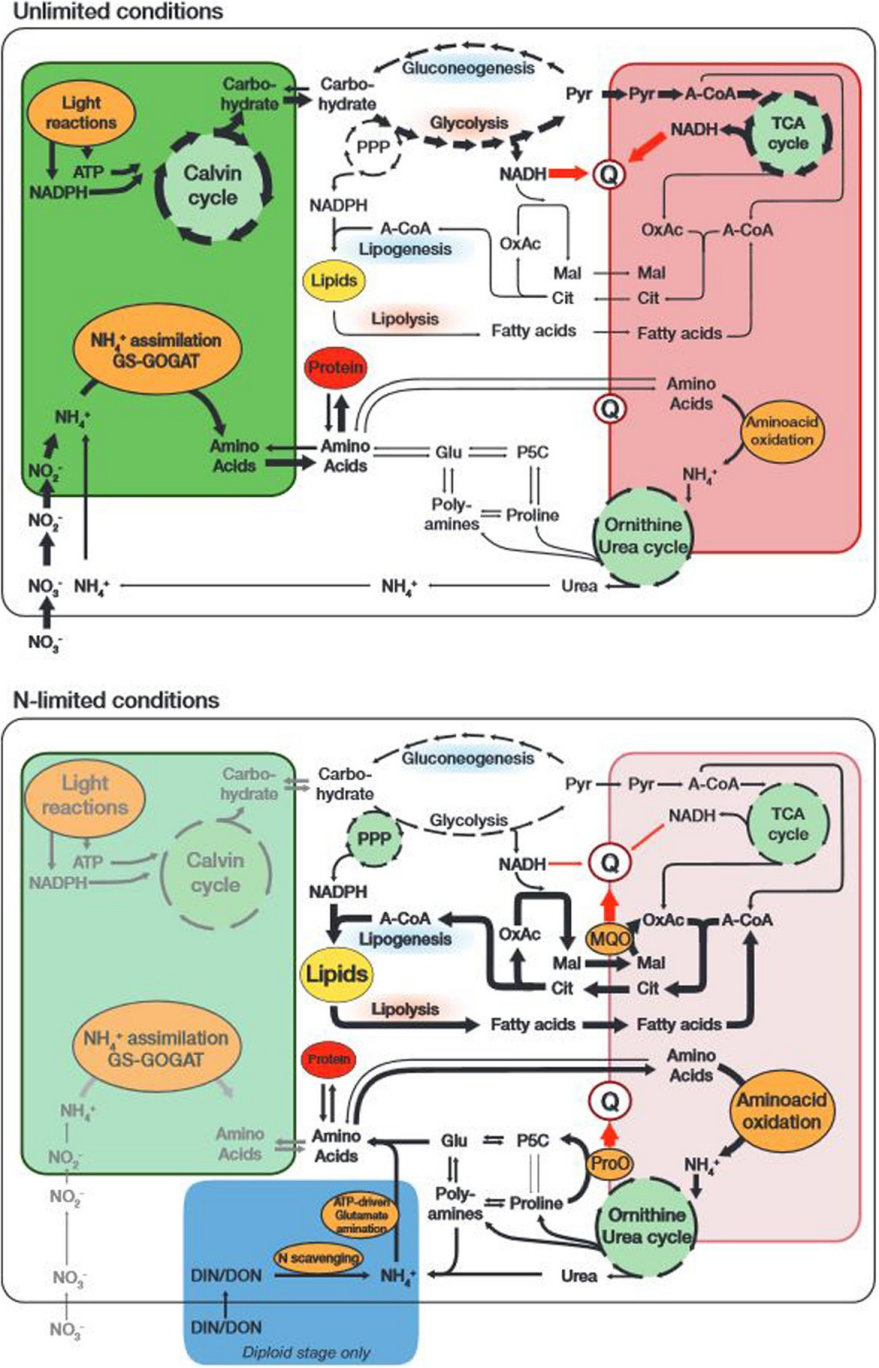
**Proposed generalized metabolic constellations in** ***Emiliania huxleyi*** **under N-replete and N-limited conditions:** **Under N-replete conditions (top), photosynthetic light reactions produce ATP and NADPH, which are consumed in the photosynthetic Calvin cycle.** After export from the plastid, the produced carbohydrates are submitted to glycolysis to produce pyruvate (Pyr), which is transported to mitochondria and burned in the tricarboxylic acid (TCA-) cycle. The NADH created in cytoplasm and mitochondrion is fed into the quinone-pool (Q) of the mitochondrial respiratory chain. Lipogenesis that relies on the shuttling of citrate and malate as well as NADPH production by the pentose-phosphate pathway (PPP) works normally. Plastidary N-assimilation via the GS-GOGAT cycle provides amino acids for protein synthesis. Under N-limited conditions (bottom), photosynthesis, i.e., carbon and energy input is minimized, so that the flux through glycolysis and the TCA cycle is reduced. Cells strongly induce the citrate-shuttle that exports acetyl-CoA into the cytoplasm and increase the relative intensity of lipid turnover (synthesis and oxidation), which provides required sinks for excess reduction equivalents. To facilitate electron input into Q under these adverse flux conditions, cells make use of a malate:quinone oxidoreductase (MQO). Mitochondrial amino acid oxidation is increased, while enzymes of the ornithine-urea-cycle (OUC) and connected reactions are throttled, putatively leading to accumulation of OUC intermediates as well as polyamines and proline. The oxidation of proline to pyrroline-5-carboxylate (P5C) by proline oxidase (ProO) also appears to be a major entryway for reductants into Q when N is scarce, and a gateway to feed stored N into the metabolism. Especially the diploid stage induces machinery to scavenge external (in)organic nitrogenous compounds (blue shade).

Under N-limitation, the diploid cells increased expression of NH_4_^+^ transporters and multiple enzymes that scavenge N from various external organic sources, e.g., azoreductase (GJ05276, GJ06383), nitropropane dioxygenase (GJ07219) or carbon-nitrogen-hydrolase (GJ13749). The ability to use external NH_4_^+^ and organic nitrogenous compounds has been demonstrated earlier and is thought to contribute to *E. huxleyi*’s broad ecological range [28]. Plastidic nitrite (NO_2_^−^) reductase (GJ07852) was down-regulated, probably due to inactivation of this organelle. No regulation was measured for NO_3_^−^ reductase, although enzyme activity has been shown earlier to cease in *E. huxleyi* under N-limitation [29]. In addition to external scavenging, the cells induced mitochondrial amino acid oxidation, i.e., catabolic reactions that efficiently cleave NH_4_^+^ from the cellular amino acids (aspartate aminotransferase/glutamate-oxaloacetate transaminase GJ19978; NADH-dependent glutamate dehydrogenase GJ00044). The data strongly suggest that the process of amino acid oxidation is biochemically succeeded by an ornithine-urea cycle (OUC; Figure 4). This cycle was long thought to facilitate *clearance* of N in metazoans, but was recently shown to play an important role as a hub for N-redistribution also in diatoms and other photosynthetic protists [30–33]. Diploid, N-limited *E. huxleyi* down-regulated most genes of the OUC (carbamoylphosphate synthetase GJ00207, GJ00711, GJ10276, GJ11368; ornithine transcarbamoylase GJ02569; argininosuccinate lyase 1 GJ04986, GJ10215; Arginase, GJ14778), which closely resembles the responses of diatoms to N-limitation [30, 31]. Furthermore, genes that connect the OUC with proline- and polyamine synthetic pathways were also found down-regulated (ornithine aminotransferase GJ10009; ornithine decarboxylase GJ00974, GJ11436; spermine/spermidine synthase GJ05786, GJ16513). This down-regulation of the OUC and the branching polyamine-synthesis indicates that overall metabolite flux is decreased. However, decreased enzyme activity could also lead to increased pool sizes of the connected metabolites, and especially with increased NH_4_^+^ input, it might be that a relatively higher fraction of cellular N is reallocated to these compounds. Such a mechanism could establish biochemical sinks that retain N from the rest of the metabolism and function as saving deposits, which are less readily accessible than amino acid pools. The osmoprotective functions of polyamines are coincidently beneficial and closely associated to various cellular stress responses in many phototrophs [30, 34–37]. Certainly, the dynamics of polyamine pools should be subject of further interrogations using metabolomic techniques.

The prominent up-regulation of proline oxidase (GJ05200), which feeds reduction equivalents into the electron transfer chain (ETC; Figure 4), suggests that proline oxidation may be a considerable contributor of ATP synthesis under N-limitation. Moreover, it may be hypothesized that proline oxidation at the same time serves as a redox-sensor: Proline is a side-metabolite of polyamine metabolism [35, 36]. Its concentration and thus reductant input into the ETC should -depending on metabolic constellations- be somewhat proportional to the metabolite load of the polyamine pools, and reflect approximately the availability of cellular N. Such a concentration-dependent mechanism may indeed be responsible for the amplification of expression patterns observed between the sampled time points (Figure 3). In this way, the OUC and the proline cycle could constitute a metabolic sensor for cellular N budgets, as similarly suspected for higher plants and metazoans [38, 39]. Interestingly, the proline oxidase is more known for its role as a highly conserved tumor suppressor that causes cell cycle arrest in the G_2_ phase and regulates apoptosis, both in plants and metazoan cells [36, 38–41]. Its prominent induction in N-limited, cell-cycle arrested *E. huxleyi* highlights that the molecular decisions, balancing growth versus cell cycle arrest or apoptosis in response to macronutrient status, appear widely and highly conserved across eukaryote lineages.

To release N from the polyamine stores, cells appear to make use of amine oxidases (GJ15345, GJ19732) and pyrroline dehydrogenase (GJ01187). To mobilize N via the OUC, cells induced a distinct argininosuccinate-lyase 2 (GJ01289) and urease (GJ22989) that appear to support the withdrawal of urea and ultimately NH_4_^+^ from the cycle. The NH_4_^+^ released to the cytoplasm appears to be salvaged by a cytosolic glutamine synthetase (GJ01535, GJ28143; [42]), which catalyzes the ATP-driven amination of glutamate and is known to be especially induced under N-limitation (Figure 4; [43]). Thus, cells appeared to replace the plastid-based N assimilation via the GS/GOGAT system [44] by a cytoplasmic NH_4_^+^ assimilation. Such a mechanism could profit from a higher relative ATP availability when protein translation is shut down.

### Carbon fluxes

The reconfigurations of the N metabolism were paralleled by a re-structuring of mitochondrial carbon fluxes. The mitochondrial aspartate/glutamate translocator (GJ05042) was up-regulated, suggesting increased export of oxaloacetate from the mitochondria in exchange for amino acids destined for oxidation. Conventionally, oxaloacetate is reduced to malate in the cytoplasm, and the malate is then shuttled back into the mitochondria, a mechanism known as the malate-aspartate-shuttle, which conveys reduction equivalents to the mitochondrial matrix. However, the counterpart to complete the shuttle, the oxoglutarate/malate carrier (GJ07712) was found down-regulated under N-limitation, an expression pattern observed previously in N-limited *E. huxleyi* [45]. To still complete the shuttle mechanism, cells induce tricarboxylate carriers (GJ03542, GJ25096) that facilitate the malate import but simultaneously export citrate from the mitochondria, ultimately delivering acetyl-CoA to the cytoplasm.

Such increased export of acetyl-CoA from the mitochondria supplies substrates for the buildup of lipids, and indeed, up-regulation of a β-ketoacyl-ACP reductase (GJ04287), and ‘very long-chain fatty acid condensing enzyme 17’ (GJ03551, GJ00296) were observed. Likewise, acyl-CoA synthetase (GJ00540), that commits fatty acids for oxidation, was down-regulated, which also should increase the relative lipid content of cells. However, a cohort of enzymes involved in fatty acid synthesis was concertedly down-regulated (acetyl-CoA carboxylase GJ16107; 3-oxoacyl-(acyl-carrier-protein) synthase GJ04276; enoyl-CoA dehydratase, GJ04137; long chain fatty acid elongase GJ11770). These rearrangements in fatty acid metabolism indicate an alteration of types and sites of lipid synthesis due to changed lipid demands and explain the often observed massive lipogenesis under N-limitation in *E. huxleyi* (Figure 4; [46, 47]). The induction of lipogenesis in N-limited diatoms likely follows similar mechanisms and cell-biological constraints, e.g. requirement for transient sinks for C and reductant [48].

The plastid localized, but nuclear encoded genes of the shikimate pathway, which generates aromatic compounds, were down-regulated (DAHP synthase GJ04247; shikimate kinase GJ05792; chorismate synthase GJ13562, GJ05023). Aromatics are crucial precursors of quinones and down-regulation of this pathway likely feeds forward on the carrying capacity of the ETC, which, indeed, greatly rearranged under N-limitation.

### Electron fluxes

The glycerol-3-phosphate shuttle that conveys cytoplasmic NADH directly into the ETC was found down-regulated (GJ21847, GJ01847, GJ21846), which might be also related to the decreased needs for glycerol-based membranelipids. Consequently, relatively more NADH is available and the reduction of oxaloacetate to malate in the context of the discussed mitochondrial shuttling likely becomes the prime catabolic sink for cytoplasmic reductant. Enzyme complexes of the respiratory chain were prominently down-regulated (e.g. NADH:ubiquinone oxidoreductase GJ12786, GJ14395; cytochrome complex GJ03828, GJ16747; Subunits of F_O_F_1_ ATP synthase, GJ00632, GJ05568), indicating that the conventional electron input of NADH into the ETC via Complex I as well as ATP synthesis in general were decreased. To compensate this, *E. huxleyi* induced a mitochondrial malate:quinone oxidoreductase (MQO, GJ00410; Figure 4). This enzyme has been characterized mainly in prokaryotes but has recently been recognized in a number of other alveolate protists [49] and appears to be absent from diatom genomes (*Thalassiosira pseudonana, Phaeodactylum tricornutum, Fragilariopsis cylindrus;*http://genome.jgi-psf.org/). MQO transfers electrons directly from malate to the quinone pool of the ETC [50] and thereby bypasses the conventional malate-dehydrogenase (MDH) of the TCA-cycle. This appears to be of strong advantage because the latter reaction has a very unfavorable standard free energy difference (Δ*G*°’ = 28.5 kJ mol^−1^): It will only take place when the TCA cycle is simultaneously removing oxaloacetate, i.e., burning carbon. In contrast, the MQO-catalyzed oxidation of malate with ubiquinone has a very favorable standard free energy difference (ΔG°’ = −55 kJ mol^−1^), allowing the organism to efficiently oxidize malate even under circumstances where a conventional MDH is unable to do so, e.g., when ratios of mitochondrial [oxaloacetate]/[malate] or [NADH]/[NAD] are high (e.g., [51]). Apparently, the observed cumulated activity of lipid turnover and MQO feeds [e^−^] into the ETC, reducing respiratory carbon loss (Figure 4). These metabolic rearrangements are likely to support efficient ATP synthesis under adverse environmental constellations, e.g., nutrient limitation.

### Haploid stage

In the haploid life-cycle stage of *E. huxleyi*, N-limitation showed significant up- and down-regulation of 1457 and 2174 features, respectively, and those responses were functionally nearly congruent with the diploid stage.

### Deactivation of protein biosynthesis

A general down-regulation of biosynthesis occurred at the level of RNA transcription and protein translation in all cellular compartments, similar to the diplont. However, overall fewer features in the haplont than in the diplont indicated down-regulation of transcriptional machinery (RNA Polymerase II, second largest subunit GJ10007), ribosomal components (Ribsomal protein L21 GJ22830) and diverse tRNA synthetases. These less pronounced regulation patterns with respect to transcription and translation are consistent with the previous suggestion that the haploid stage administrates its proteome more on posttranslational bases rather than through transcriptional and translational regulation [19].

### Deactivation of plastids

Data indicated a down-regulation of photosynthetic light reactions, involving mostly the genetic machinery that was observed in the diploid stage, e.g., photosystem components (PS II reaction center protein GJ09667), light harvesting antennae (light harvesting proteins, GJ19276, GJ18462), enzymes of the xanthophyll cycle (zeaxanthin epoxidase GJ 22205, GJ00453, GJ05220, violaxanthin deepoxidase GJ02541, GJ07458), as well as chlorophyll synthesis (Protochlorophyllid reductase GJ01226, Mg-chelatase (GJ00007). Also, the decreased expression levels of plastidic translocators for glucose-6-phosphate/phosphate and phosphoenolpyruvate/phosphate (GJ03350, GJ07072, GJ01768) support the notion that, like in the diploid life-cycle stage, plastid activity was reduced and consequently, metabolic flux between plastid and cytoplasm was decreased under N-limitation.

### Reconstellation of nitrogen metabolism

The effects of N-limitation on the haploid metabolism shared common features with the diplont response, e.g., the down-regulation of the synthesis of key amino acids, purines and pyrimidines. The OUC was, like in the diploid stage, down-regulated in key reactions (carbamoylphosphate synthase GJ11368, GJ00711; ornithine transporter GJ27211; argininosuccinate synthase GJ01516) and also in peripheral reactions that divert intermediates towards proline or polyamine synthesis (ornithine aminotransferase GJ00647; ornithine decarboxylase GJ06908). These regulation patterns indicate that the haploid stage applies the same biochemical strategy of using OUC cycle intermediates and their derivatives as a cellular backup for N.

Clearly visible in the haploid stage was the down-regulation of the plastidary GS-GOGAT system, which conventionally assimilates inorganic N (glutamine-synthetase GJ00014, GJ09184, GJ12379; glutamate-synthase GJ12602), as well as the machinery for assimilation of oxidized inorganic nitrogen sources (NO_2_^−^ transporter GJ07216; NO_3_^−^ transporter GJ16902, GJ15318, GJ00825; NO_2_^−^ reductase GJ21771, GJ00725; NO_3_^−^ reductase GJ04471, GJ23958, GJ09099). The down-regulation of NO_3_^−^ reductase under N-limitation has been observed earlier in diploid *E. huxleyi* cultures [29, 52] and in N-limited diatoms [31], and has been attributed to a substrate-sensing regulatory system that enables transcription of NO_3_^−^ related genes only in the presence of NO_3_^−^ [53]. Unlike the diplont, the haploid stage did not induce an ATP-driven cytoplasmic glutamate amination to salvage NH_4_^+^ liberated from internal amino acid turnover. Likewise, no expression changes in the machinery for the uptake of dissolved (in)organic N was seen in the haplont. One explanation may be that salvaging of dissolved (in)organic nitrogenous compounds may be constitutively expressed in the haploid stage, so that no expression changes could be seen.

### Carbon fluxes

In the haploid response to N-limitation, enzymes of fatty acid synthesis were concertedly down-regulated (acetyl-CoA carboxylase GJ00002, GJ00064; malonyl -ACP transacylase GJ05975, GJ03112; 3-oxoacyl-ACP-synthase I and II GJ01429; polyunsaturated fatty acid specific elongases GJ04003, GJ11347), while enzymes involved in lipid oxidation were up-regulated (acyl-CoA dehydrogenase GJ07324; hydroxyacyl-CoA dehydrogenase GJ00214, GJ00215,GJ03356). This clearly suggests a prioritization of lipid oxidation, i.e., a provision of C in form of acetyl-CoA. In line with this was the up-regulation of the carnitine shuttle, which imports fatty acids to the mitochondrial site of oxidation (L-carnitine dehydratase GJ20083; carnitine/acylcarnitine translocator GJ11395) and the classical marker enzymes isocitrate lyase (GJ00349) and malate synthase (GJ00164). These enzymes skip parts of the TCA cycle and instead reroute carbon into a glyoxylate cycle, so that acetyl-CoA derived from fatty acid oxidation is used to produce succinate and malate. This bypass feeds formerly immobilized carbon back into the primary metabolism. The down-regulation of the mitochondrial tricarboxylate carrier (GJ03542) was opposite to that of the diploid stage, suggesting that in the haploid stage, different gene sets for lipogenesis and lipolysis operate that are more strictly counter-regulated, whereas in the diplont these systems can run to a large extent simultaneously. However, under N-limitation the haploid stage induced Na^+^/dicarboxylate carriers (GJ11524, GJ06704) and also a malate/oxaloacetate translocator (GJ27728). Thus, the haplont uses different transporters, but the resulting transporter constellation may however be well able to facilitate traffic of metabolites similar to the diploid stage. It appears that the abundance and relative activity of different mitochondrial shuttle systems critically determines the direction of flow of C and [e^−^], and in consequence also N. These regulation patterns are in line with previous transcriptomic data suggesting that intense fatty acid turnover is a special property of the haploid life-cycle stage of *E. huxleyi* [19, 54].

### Electron fluxes

Data suggests that, like in diploid cells, the decreased photosynthetic input of C and reductants is paralleled by the same adjustments of mitochondrial connectivity and electron sinks: The down-regulation of the glycerol-3-phosphate shuttle (GJ01847, GJ07067, GJ02661), the NADH:ubiquinone oxidoreductase (GJ12786, GJ05219, GJ00231), the cytochrome complex (GJ03931, GJ25391, GJ22387) and multiple subunits of F_O_F_1_ ATP synthase (GJ26307, GJ05568, GJ00632) indicated that under N-limitation, conventional ATP generation was decreased and cellular energy budgets were considerably reduced. Succinate dehydrogenase, however, was up-regulated (GJ00350, GJ27868, GJ05263), possibly due to increased activity of the glyoxylate cycle, of which succinate is a direct product. Also in the haplont, proline oxidase (GJ05200) was prominently up-regulated, supporting the notion that OUC and proline oxidation constitute an important sensing mechanisms that is a crucial element in the cellular N-budgeting. Also, like cells of the diploid life-cycle stage, the haplont induced mitochondrial MQO to maintain electron flux through the respiratory chain even under adverse environmental conditions.

### Ecophysiological significance of nitrogen-limitation responses

The obtained transcriptomic data well support the flux modes of N through protistan metabolism as they were previously hypothesized by [30] and [31]. Also in *E. huxleyi*, the OUC appears to be a distributional hub for N that balances carbon assimilation and breakdown with available cellular nitrogen budgets. This balancing may be achieved because N and C metabolism rely on and respond to common substrates [31, 32, 55]. The presence of OUC genes also in dinoflagellates [33] suggests that the described metabolic responses satisfy biochemical requirements which are generally imposed on eukaryotic cells in nutrient-scarce environments. Here we show the first evidence that *E. huxleyi* applies an MQO, an interesting genetic feature among eukaryotic phytoplankton, which seems to enable organisms to drive efficient ATP synthesis with reduced respiratory carbon loss. This enzyme might contribute to *E. huxleyi*’s ability to inhabit very divergent environments [7, 8, 56]. The profound regulation patterns we observed in response to N limitation are not recognizable in the response to sulfate deficiency as presented by Bochenek et al. [57]. Under sulfate deprivation, cell division was slowed down, but growth in terms of biomass production was still high, as can be derived from the strongly increased cell volumes. This may be the reason why under sulfate deficiency there was no indication of a comparable large-scale metabolic re-configurations as seen under N-limitation.

The haploid cells, which might function as a ‘seeding population’ in the aftermath of viral bloom termination [17], showed less transcriptomic responses globally, which paralleled the lower increase in particulate C:N ratio under N-limitation in haploid compared to diploid cells (Additional file 2: Figure S2). Haploid cells apparently did not specifically regulate genes for scavenging external N sources when confronted with N-limitation. This may either be due to a constitutive expression of N scavenging machinery or perhaps because the haplont does not rely on these genes at all, being more adapted for ‘post-bloom’ environments, in which waters may contain higher amounts of organic nutrients released from decaying phytoplankton. As both life-cycle stages appear to have mixotrophic capacities [19], in the hypothesized natural scenario, haplonts might generally be able to rely more on external dissolved or particulate sources of N. These diverging morphological and physiological properties support the notion that the stages are specialized to occupy distinct ecological niches.

## Conclusions

The two life-cycle stages showed distinct physiological responses to N-limitation, reflected also in very different sets of genes responding in both phases and a lower overall transcriptomic response in the haploid phase compared to the diploid phase. These results strongly support the theory that the haploid and diploid life-cycle stages are evolutionarily shaped for the distinct ecological niches they occupy. Despite strong physiological differences between the responses of each stage, fundamental functional similarities emerged from the data: Under N-limitation, both life-cycle stages shut down growth and biosynthetic activity. Cells switch into a ‘low-throughput metabolism’ (Figure 4) in which C fixation and respiration are minimized and cellular N is efficiently salvaged. The redistribution of N among internal pools of polyamines and the mobilization via the OUC resembles metabolic behavior documented in other protists. This suggests functional commonalities across distant phylogenetic clades. The OUC and proline oxidation might play a central role in coordinating and integrating the cellular fluxes of C, N and reductants in most, if not all eukaryotes. The deployment of an MQO that supports ATP synthesis under limitation appears to be a principal and advantageous component of *E. huxleyi*’s cellular energy management. Its enduring photosynthesis, parsimonious N budgeting and efficient ATP generation in situations of limitation may be hallmarks of the success of the *E. huxleyi* diplont in contemporary oceans.

## Methods

### Culturing, growth and fluorometry

Diploid and haploid cells of *E. huxleyi* (Strains RCC 1216 and RCC1217, respectively, both obtained from the Roscoff Culture Collection) were cultured in 5 L Schott borosilicate bottles (biological triplication) containing 0.2 μm filtered North Sea seawater medium (salinity 32). Cultures were grown at 15°C under a light:dark cycle of 16:8 hours under light intensities of 250 ± 25 μmol photons m^−2^ s^−1^ (FQ 54 W/965HO daylight lamps; OSRAM, Munich, Germany), as measured with a datalogger (LI-1400; Li-Cor, Lincoln, USA) equipped with a 4*π*-sensor (Walz, Effeltrich, Germany). The control medium (N-replete) was enriched with vitamins and trace-metals (F/2 medium; Guillard & Ryther 1962). Nitrate (NO_3_^−^) and phosphate (PO_4_^3−^) were added in concentrations of 100 and 6.25 μmol kg^−1^. The ‘N-limited’ medium was supplemented with vitamins, trace-metals and PO_4_^3−^ only. Prior to the experiment, cells were pre-acclimated to the experimental settings for 10 days in dilute batch cultures, maintaining exponential growth. Over the duration of the experiment (13 days), medium was purged with humidified air (flow-rate 130 ± 20 mL min^−1^) to counteract cell sedimentation and to replenish dissolved inorganic carbon (DIC). In addition, bottles were manually moved several times a day to enhance suspension of cells. The concentrations of haploid and diploid cells, growing under ‘control’ and ‘N-limited’ conditions, were assayed on a daily bases using a Multi-Sizer III hemocytometer (Beckman-Coulter, Fullerton, USA). Specific growth rates were calculated from daily increments following the equation μ = (ln(c_1_)-ln(c_0_)) Δt^−1^, where c_0_ and c_1_ are the cell concentrations at two time points and Δt is the time interval. To follow inorganic carbon chemistry, total alkalinity (TA) was calculated from linear Gran-titration plots [58], obtained from automated titration of filtered culture medium (TitroLine alpha plus, Schott, Mainz, Germany). Concentrations of DIC were measured colorimetrically [59], using a QUAATRO autoanalyzer (Seal Analytical, Norderstedt, Germany. CO_2_ and pH were calculated using CO_2_SYS [60] and were based on measurements of DIC, TA, temperature, salinity and PO_4_^3−^. Dissociation constants for carbonic acid and sulfuric acid were used from [61] and [62].

For elemental analyses of particulate organic C and N (POC, PON), cultures were gently filtered onto pre-combusted GF/F filters (12 h, 500°C, 1.2 *μ*m pore size; Whatman). Prior to the determination of POC contents, respective filters were acidified with 200 *μ*L 0.2 mol L^−1^ HCl (Merck) to remove any calcite. Analyses were carried out using an EuroVector CHNS-O elemental analyzer (EuroEA, Milano, Italy). Cellular PIC quotas were assessed using the ICP-MS method developed by [63].

To assess photosynthetic viability of the cells, the ratios of variable:maximal fluorescence (F_v_/F_m_) were assessed using a fast-induction relaxation fluorometer (FIRe, Satlantic, Halifax, Canada).

### Microarray hybridization

Haploid and diploid cultures were sampled at two time points that reflect ‘early limitation’ (days 9 and 10 in haploid and diploid cultures, respectively) and ‘full limitation’ (days 11 and 12 in haploid and diploid cultures, respectively). As reference points, RNA was sampled from haploid and diploid cultures grown in control medium (N-replete) at days 6 and 7, reflecting unlimited exponential growth in dilute culture. The microarray-based transcriptome screening followed the protocol from [24]. In brief, ~1.5*10^7^ cells were harvested and disrupted in a beadmill. The extracted RNA (Qiagen RNeasy) was DNAse treated and enriched by ultrafiltration (MicroCon YM 30 columns, Millipore). Integrity of isolated RNA was verified using a Bioanalyzer 2100 (Agilent, Waldbronn, Germany). RNA Spike-In Mix (Agilent, p/n 5188–5279) was added to 250 ng RNA of the samples as a benchmark of hybridization performance prior to cDNA synthesis and cRNA synthesis/labeling reactions (Two-color low RNA Input fluorescent linear amplification kit, Agilent, p/n 5184–3523). 750 ng of each Cy-3 and Cy-5 labeled cRNA were hybridized onto 2*105 K *Emiliania huxleyi* custom-built microarrays (Agilent, Design# 022065). Three microarray probes were designed (i.e., on-chip technical replication) based on 39.091 Sanger EST sequences obtained from strains RCC1216 and RCC 1217 [16] and 72.513 Sanger EST sequences from CCMP1516 [7] that were assembled into 28670 transcript clusters using the whole genome assembly of *Emiliania huxleyi* CCMP1516 [7] as a reference. This microarray design has been used in prior studies and probe-sequences and chip layout are openly accessible from the ArrayExpress database (http://www.ebi.ac.uk/arrayexpress/arrays/A-MEXP-2177). The original reference transcript sequences are available as supporting information in FASTA format (Additional file 6: S6). Hybridization was performed following the Two-Color Microarray-based Gene Expression Analysis protocol (Agilent, p/n 5188–5242). Arrays were immediately scanned after hybridization with a G2505C microarray scanner (Agilent) using standard photomultiplier tube settings and 5 μm scan resolution. In the microarray hybridizations, every biological replicate was hybridized against the same common control baseline (i.e., pooled control RNA from all treatments) to minimize hybridization biases. Treatment-vs.-treatment expression ratios were then calculated from the single treatment-vs.-control expression ratios. All further reported expression ratios are relative to the control, i.e., unlimited exponential growth in the N-replete treatment.

### Data treatment

Raw data was extracted with Feature Extraction Software version 9.0 (Agilent). Analysis was performed using GeneSpring 11 (Agilent). LOWESS-normalized data were submitted to the MIAMExpress database hosted by the European Bioinformatics Institute (EBI; http://www.ebi.ac.uk/arrayexpress; accession code E-MTAB-2274). The hybridization results of the biological triplicates (i.e., treatment-vs.-treatment expression ratios) were evaluated using multiple comparison tests (ANOVA) followed by a Benjamini-Hochberg false-discovery-rate filtering procedure [64] provided in the Genespring 11 software package. Regulation was called significant when probe-specific corrected p-values were <0.05. The dataset was then reduced to only those probes, which detected expression changes in response to the N-limitation treatment by more than 1.5-fold. When a divergent regulation was reported, i.e., probes for the same transcript cluster indicating regulation in opposite directions, the respective probe set was as a whole excluded from further analyses (<15 probe sets per hybridization). In case that only one out of three probes reported significant differential expression but two probes reported unaltered expression, the respective probe sets were as well excluded from further analyses to further increase the confidence level of results (300–700 probe sets per hybridization). The remaining probe sets were then merged, and are in the manuscript reported as significantly regulated features representing one transcript cluster. For completeness, the number of hit probes and the average fold-change values and obtained (corrected) p-values are reported for every transcript cluster.

### Data annotation

Significantly regulated clusters were assigned to an annotation table. This table was generated using *BLASTn* similarity searches, in which the 28670 transcript clusters were aligned with the ‘Emihu1_best_transcripts’ database provided by the Joint Genome Institute (JGI). After excluding alignments with an e-value <10^-5^, the two best aligning, but different transcript models were implemented into the annotation table. This allowed the assignment of ~21740 investigated transcript clusters to models existing in the JGI *E. huxleyi* gene catalog. Assigned JGI model IDs were then aligned with the ‘best gene-model’ predictions, based on similarity to eukaryotic orthologous genes (KOG; provided by the JGI). This KOG-database harbors functional information on ~11930 different *E. huxleyi* models in the JGI catalog. Additionally, generic information on the investigated transcript clusters was obtained by Blast2GO (B2G) queries ([65]; e-value cutoff at >10^-6^), and the GO information was augmented to the annotation. The final transcriptome screening involved ~10.000 *E. huxleyi* transcripts with a confidently predicted function. The integrity and validity of gene models of interest that are discussed in the text were reconfirmed by model inspection in the JGI draft genome database and by BLAST searches. In the text, exemplary transcripts of interest are notated with their numerical cluster identifiers.

### Availability of supporting data

Treated data sets, figures and tables backing up the results of this article are available as Supporting Information. The microarray raw data set supporting the results of this article is deposited and publicly available at the EBI Arrayexpress repository: http://www.ebi.ac.uk/arrayexpress; accession code E-MTAB-2274.

## Electronic supplementary material

Additional file 1: Figure S1: CO_2_ concentrations **(a)** and pH values **(b)** over the course of the experiment, as calculated from TA and DIC. Color coding follows Figure 1. Error bars denote 1 SD (n = 3). (PDF 20 KB)

Additional file 2: Figure S2: Cellular elemental quotas and ratios over the course of the experiment. Color coding follows Figure 1; **(a)** Particulate organic carbon (POC) quota; **(b)** Particulate organic nitrogen (PON) quota; **(c)** Atomic ratio of POC:PON; **(d)** Particulate inorganic carbon (PIC) quota; **(e)** Atomic ratio of PIC:POC. Error bars denote 1 SD (n = 3). (PDF 352 KB)

Additional file 3: Figure S3: 4-way Venn diagram representation of the life-cycle stages’ responses to N-limitation. Italic and underlined black numbers indicate significantly up- and down-regulated transcript clusters, respectively; red numbers indicate counter-regulated transcript clusters. (PDF 81 KB)

Additional file 4: Spreadsheet S4: Transcript clusters regulated in response to N-limitation in the haploid and diploid life-cycle stages of *Emiliania huxleyi* at time points of early and full limitation. (XLSX 3 MB)

Additional file 5: Spreadsheet S5: List of only those significantly regulated transcript clusters that were mentioned in the text. Clusters are responsive to N-limitation in the haploid and diploid life-cycle stages of *Emiliania huxleyi* at time points of early and full limitation. (XLSX 106 KB)

Additional file 6: FASTA file S6: Original transcript clusters derived from the genome sequences of *Emiliania huxleyi* CCMP1516 (JGI, [7]) and EST sequences from strains RCC1216 and RCC 1217 ([16]) used for construction of microarray probes and annotation. (ZIP 7 MB)

## Abbreviations

C: Carbon
N: Nitrogen
P: Phosphorus
NO_3_^−^: Nitrate
PO_4_^3−^: Phopshate
TA: Total alkalinity
DIC: Dissolved inorganic carbon
F_v_/F_m_: Ratio of variable:maximal fluorescence of photosystem II
ATP: Adenosinetriphosphate
NO_2_^−^: Nitrite
OUC: Ornithine-urea cycle
NH_4_^+^: Ammonia
ETC: Electron-transfer chain
NAD/NADH: Nicotinamideadeninedinucleotide, oxidized/reduced
NADP/NADPH: Nicotinamideadeninedinucleotidephosphate, oxidized/reduced
MQO: Malate:quinone oxidoreductase
TCA cycle: Tricarboxylic acid cycle.
