# Supplementary figures and images for: Emiliania huxleyi endures N-limitation with an efficient metabolic budgeting and effective ATP synthesis
Source: BMC Genomics. 2014 Dec 2;15(1):1051. doi: 10.1186/1471-2164-15-1051 (PMC4301891; doi:10.1186/1471-2164-15-1051)

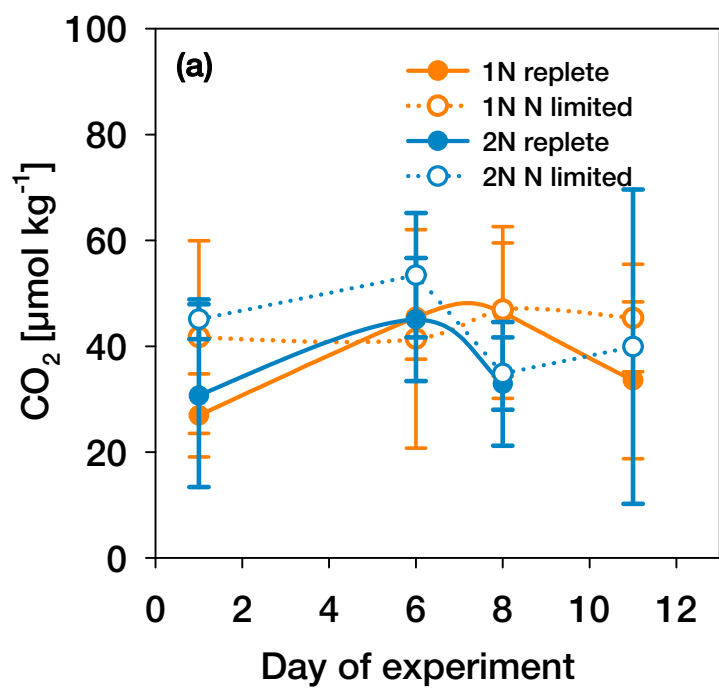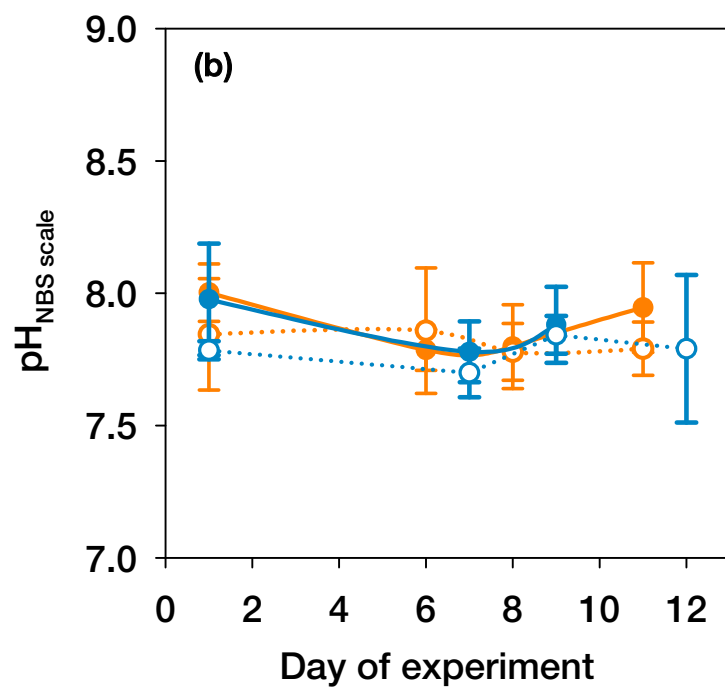

Supplement: Supplementary file 1 — Additional file 1: Figure S1: CO2 concentrations (a) and pH values (b) over the course of the experiment, as calculated from TA and DIC. Color coding follows Figure 1. Error bars denote 1 SD (n = 3). (PDF 20 KB) [file 12864_2014_6789_MOESM1_ESM.pdf]

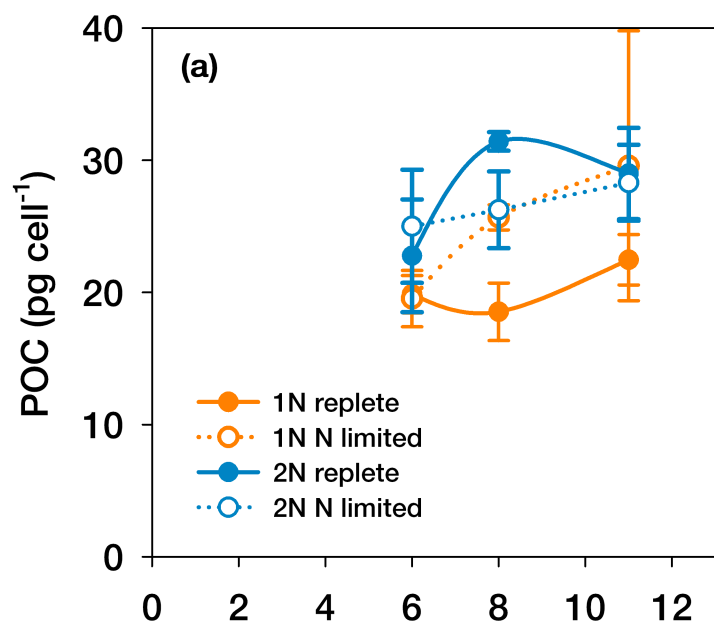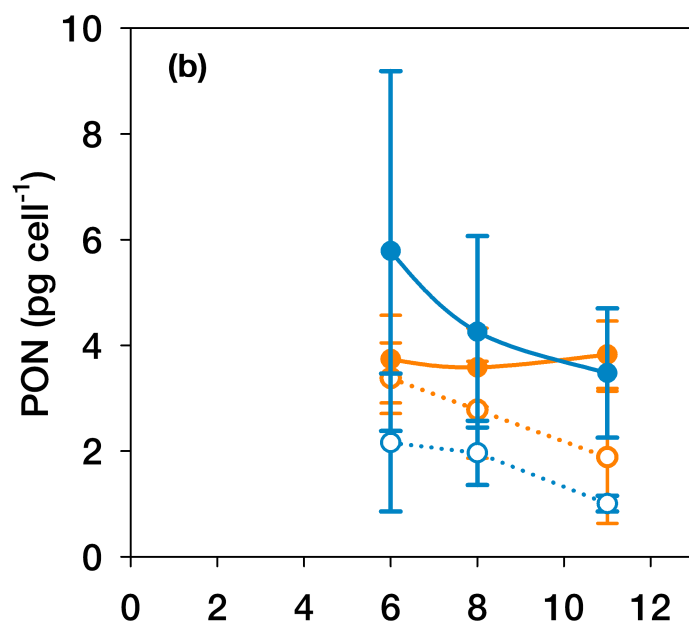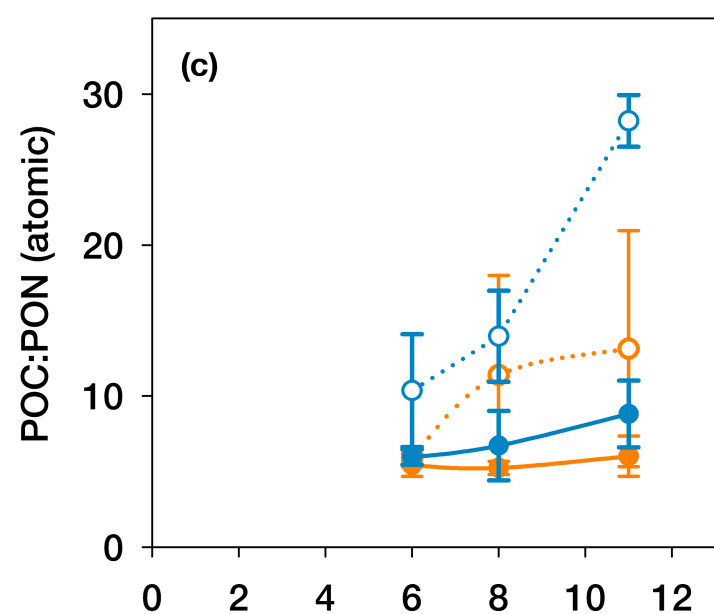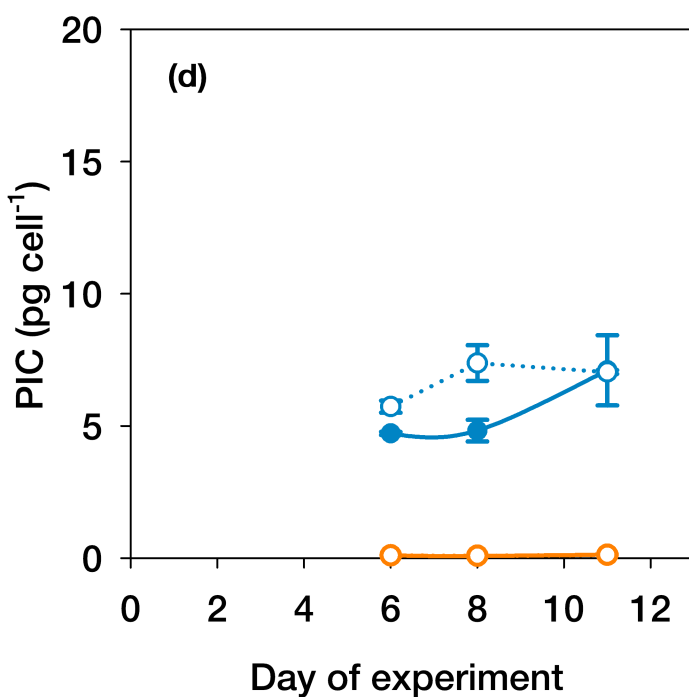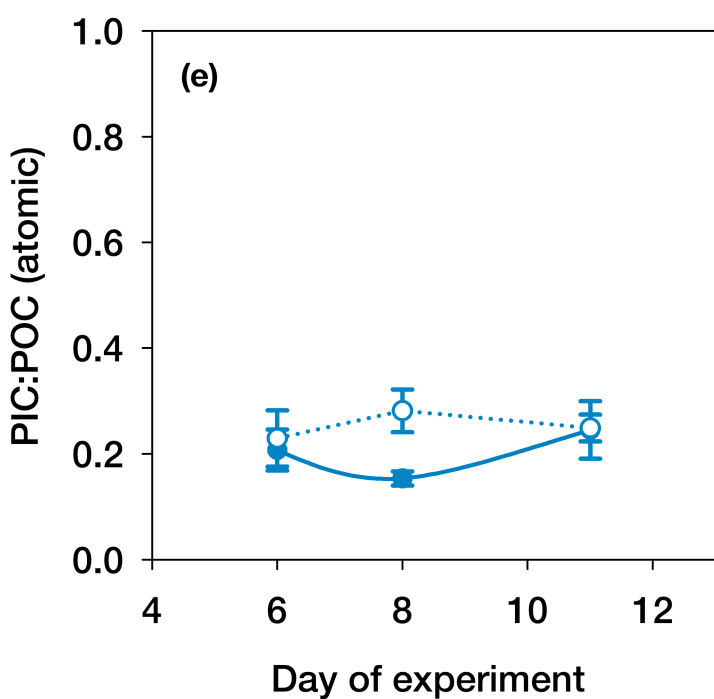

Supplement: Supplementary file 2 — Additional file 2: Figure S2: Cellular elemental quotas and ratios over the course of the experiment. Color coding follows Figure 1; (a) Particulate organic carbon (POC) quota; (b) Particulate organic nitrogen (PON) quota; (c) Atomic ratio of POC:PON; (d) Particulate inorganic carbon (PIC) quota; (e) Atomic ratio of PIC:POC. Error bars denote 1 SD (n = 3). (PDF 352 KB) [file 12864_2014_6789_MOESM2_ESM.pdf]

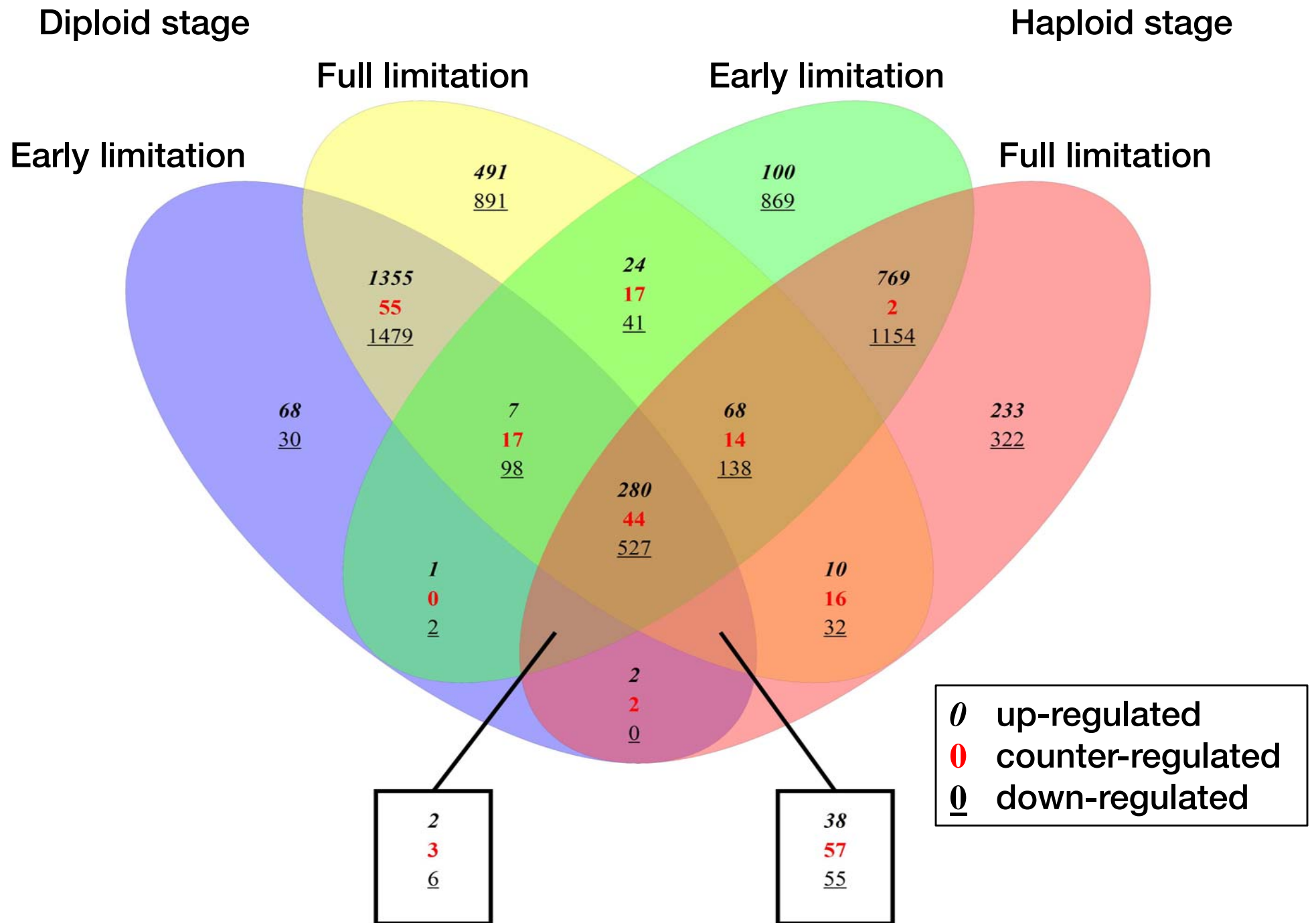

Supplement: Supplementary file 3 — Additional file 3: Figure S3: 4-way Venn diagram representation of the life-cycle stages’ responses to N-limitation. Italic and underlined black numbers indicate significantly up- and down-regulated transcript clusters, respectively; red numbers indicate counter-regulated transcript clusters. (PDF 81 KB) [file 12864_2014_6789_MOESM3_ESM.pdf]
